# Pneumatic Vitreolysis for the Treatment of Vitreomacular Traction Syndrome

**DOI:** 10.4274/tjo.galenos.2019.00400

**Published:** 2019-09-03

**Authors:** Hüseyin Baran Özdemir, Şengül Özdek, Murat Hasanreisoğlu

**Affiliations:** 1 University of Health Sciences, Ulucanlar Eye Training and Research Hospital, Ophthalmology Clinic, Ankara, Turkey; 2 Gazi University Faculty of Medicine, Department of Ophthalmology, Ankara, Turkey

**Keywords:** Vitreomacular traction, macular hole, pneumatic vitreolysis, SF6, C3F8

## Abstract

**Objectives:**

To evaluate the posterior vitreous release rates after a single injection of expansile gas in patients with vitreomacular traction (VMT) syndrome with or without associated full-thickness macular hole (FTMH).

**Materials and Methods:**

Thirteen eyes of 12 consecutive patients with VMT (11 eyes) or VMT+FTMH (2 eyes) were reviewed retrospectively. Intravitreal injection of 0.3 mL of pure sulfur hexafluoride (SF6) (9 eyes) or perfluoropropane (C3F8) (4 eyes) was performed. Bobbing the head forward and backward similar to ‘drinking bird’ head movements was instructed until VMT release. Full ophthalmic examination and optical coherence tomography was performed at each visit.

**Results:**

VMT was released in all patients (100%) and mean release time was 5.2 days (1-19 days). Macular hole closure was not achieved in either of the two eyes with FTMH. Mean central subfield thickness decreased significantly from 361 μm to 263 μm (p=0.007). The mean pretreatment visual acuity was 0.44 LogMAR, which significantly improved to 0.25 LogMAR at the last visit (p=0.003). One of 13 eyes had retinal tear after the procedure which was successfully treated with laser retinopexy. Gas migration to the anterior chamber occurred in one patient. No other complications were observed.

**Conclusion:**

Pneumatic vitreolysis with C3F8 and SF6 gases is a relatively safe, low-cost, and minimally invasive treatment modality for VMT. However, FTMH closure could not be achieved with pneumatic vitreolysis.

## Introduction

The natural course of posterior vitreous detachment (PVD) begins at the perifoveal retina and extends, in order, to the superior, temporal mid-periphery, fovea, inferior mid-periphery, and finally the optic disc margin, resulting in complete PVD.^1^ Abnormal vitreomacular adhesions cause incomplete PVD, which may in turn induce vitreomacular traction (VMT).^2^ Patients with VMT experience visual disturbances such as loss of vision, metamorphopsia, and central scotoma with distortion of the fovea.^3^ VMT is classified according to the size of vitreomacular adhesion (VMA) (focal ≤1500 µm and broad >1500 µm) and the presence of concurrent retinal pathology (isolated or not).^4^ VMT is thought to provoke cystoid macular edema, macular hole, epiretinal membrane (ERM), diabetic macular edema, and neovascular age-related macular degeneration.^5,6,7,8,9^

The initial approach to VMT is a period of observation in most patients.^10^ Wu et al.^11^ reported that VMT released spontaneously only in 21.4% of eyes, with increase in BCVA from 0.4 to 0.2 logMAR. The Pan-American Collaborative Retina Study Group indicated that observation can be recommended to selected patients. Although pars plana vitrectomy (PPV) is one of the best options for symptomatic VMT, it involves risks such as cataract formation, retinal tears, and endophthalmitis.^12,13^ Ocriplasmin (Jetrea; Thrombogenics, Inc, Iselin, NJ) was approved in 2012 by the Food and Drug Administration and came into the market for pharmacological vitreolysis, which is a less invasive intervention than PPV.^14,15^ However, VMT release rates were only about 40%. Moreover, since it is relatively expensive and may cause side effects like transient visual loss, lens subluxation, electroretinogram changes, ellipsoid zone changes, retinal breaks, and dyschromatopsias, it is far from being an ideal solution for VMT.^16,17^

Previous studies of the efficacy of intravitreal gas bubble for stage 1 and 2 macular holes yielded promising results.^18,19^ Chan et al.^18^ were the first to describe the technique of pneumatic vitreolysis (PV) in 1995. They used 0.3 mL of perfluoropropane (C3F8) and asked patients to stay in face-down position for at least 8 to 10 hours in a 24-hour period. They reported induction of PVD in 18 of 19 patients and closure of full-thickness macular hole (FTMH; Gass stage 2) in 3 of 6 patients. Ochoa-Contreras et al.^20^ demonstrated induction of PVD using intravitreal injection of sulfur hexafluoride (SF6) gas in nonproliferative diabetic retinopathy cases. Rodrigues et al.^21^ reported their PV results in VMT patients using spectral-domain optical coherence tomography (SD-OCT) and found that VMT was released in 40% and 60% of the eyes with C3F8 at 1 month and 6 months, respectively. Steinle et al.^22^ suggested the “drinking bird” maneuver to increase VMT release rates and reported successful release of VMT in 25 of 30 patients (83%).

In the present study, we aimed to evaluate the efficacy of intravitreal pure SF6 and C3F8 gas injections followed by “drinking bird” head movements for the treatment of symptomatic VMT syndrome and FTMH.

## Materials and Methods

This retrospective, single-center study includes a case series of 13 eyes of 12 patients who underwent PV to release VMT between January 2016 and May 2018. The study was approved by the Ethics Committee of Ankara Numune Training and Research Hospital (no: E-18-2266). Treatments were done by two surgeons (S.O., M.H.). All patients underwent standard ophthalmologic examination including Snellen visual acuity, anterior and posterior segment biomicroscopy, tonometry and spectral-domain optical coherence tomography (Spectralis HRA-OCT, Heidelberg Engineering, Heidelberg, Germany). Informed consent was obtained from all patients before the procedure. This study was performed in compliance with the Declaration of Helsinki.

All patients were symptomatic either with impaired vision or metamorphopsia and had been observed for spontaneous release for at least 3 months before the intervention. VMT with or without macular hole was defined by OCT criteria published previously by the International Vitreomacular Traction Study Group.^1^ OCT scans were obtained by the same experienced technician. Central subfield thickness (CST) was measured using the built-in retinal mapping software and corrected manually if measurement errors were detected. Horizontal vitreomacular adherence (HVMA) and macular hole size were measured manually with built-in calipers.

The procedure was performed under topical anesthesia (Proparacaine, Alcaine, Alcon, Fort Worth, TX). Povidone-iodine, eyelid speculum, and 30-gauge needle with a 1 mL syringe were used for injection. Intravitreal injection of 0.3-0.4 mL of pure SF6 or C3F8 was performed through the pars plana following a prophylactic limbal paracentesis to soften the eye. Intraocular pressure, vision, and central retinal artery perfusion were evaluated after the procedure. Patients were instructed to perform “drinking bird” head movements by bobbing their head from an upright to a face-down position 10 to 20 times every 30 minutes until VMT release for the first week after gas injection. Patients were seen daily in the first postoperative week, then weekly until VMT release in the first month, and at 3-month intervals thereafter, which could be modified according to the surgeon’s preferences and patient’s availability. OCT was performed in all visits. Additional examinations were done as needed. After VMT release was detected, FTMH patients were instructed to stay in face-down position for a week, while phakic patients were instructed to avoid supine position until resorption of the gas to prevent cataract formation.

Primary outcome measures were time to VMT release, changes in CST in OCT, and visual acuity. Secondary outcome measure was macular hole closure for patients with associated FTMH.

### Statistical Analysis

Statistical analyses were performed with SPSS 22.0 (IBM, Armonk, NY, USA). Snellen visual acuity was converted to logMAR. Wilcoxon signed-rank test was used to compare results.

## Results

Patient demographics, additional ocular pathologies, and baseline and post-treatment ophthalmological findings are presented in Table 1. There were 4 male and 8 female patients in the study. The mean age was 67.0 years (range: 51-87 years). The mean time between appearance of symptoms and PV was 3.85 months (range: 3-6 months). The mean follow-up time was 11.2 months (range: 2-25 months). Two eyes of 2 patients had small FTMH with VMT, 11 eyes of 10 patients had only VMT. Three of 13 eyes were pseudophakic (23.1%). The mean CST was 361 µm (range: 253-550 µm) and the mean HVMA was 369 µm (range: 64-630 µm). The diameter of macular hole was 160 µm in the first patient and 240 µm in the second patient. Pretreatment visual acuities ranged between 20/200 and 20/32 in eyes with VMT.

**Figure 1:**
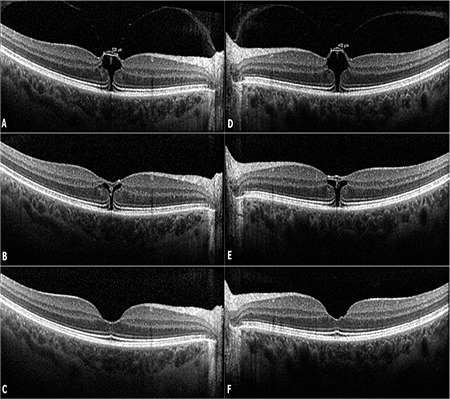
A 51-year-old woman (patient 6) presented with a complaint of blurred vision in both eyes. Snellen visual acuity was 0.63 and vitreomacular traction (VMT) was detected on spectral domain optical coherence tomography in the right (A) and left (D) eyes. Pneumatic vitreolysis was performed on the right eye first and VMT release was observed on day 3 (B). The same procedure was performed on the left eye and resulted in VMT release within 2 days (E). Snellen visual acuity increased to 0.9 in the right (C) and left (F) eyes within a month

**Figure 2:**
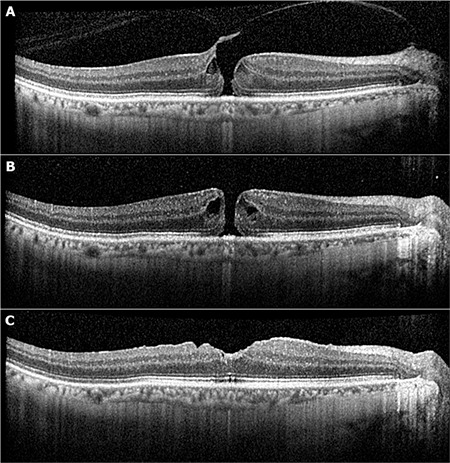
A 58-year-old man (patient 1) presented with complaint of metamorphosia involving his right eye. Snellen visual acuity was 0.5 with -6.75 D spectacle correction and there was small full-thickness macular hole (160 μm) with vitreomacular traction (VMT) on spectral domain optical coherence tomography (A). Pneumatic vitreolysis with pure C3F8 resulted in VMT release on postoperative day 4. However, a horseshoe tear was detected in the inferior equatorial retina and a laser retinopexy was performed. The patient was instructed to stay in face-down position for a week and followed-up for 45 days but the macular hole persisted (B). Macular hole closure could only be achieved after pars plana vitrectomy and final Snellen visual acuity was 0.6 (C)

VMT was released in all eyes, with a mean release time of 5.2 days (range: 1-19 days) (Figure 1). VMT was released in both of the eyes with FTMH but the holes did not close (Figure 2). Both of those eyes underwent pars plana vitrectomy which resulted in closure of the hole. The mean CST was 361 µm preoperatively, which decreased to 260 µm (range: 160-524 µm) and the difference was statistically significant (Wilcoxon signed-rank test, p=0.007). The mean LogMAR visual acuity was 0.44 at baseline and improved significantly to 0.25 (Wilcoxon signed-rank test, p=0.003).

**Figure 3:**
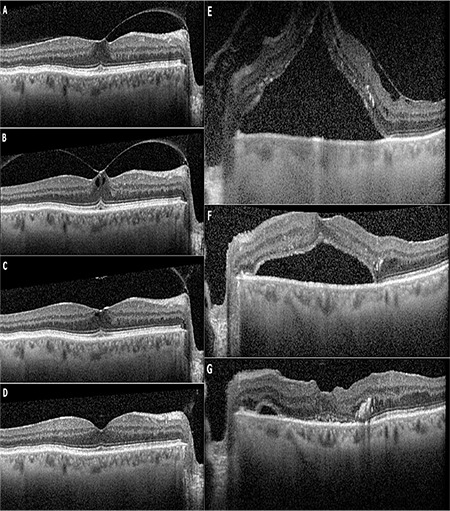
A 72-year-old patient (patient 5) who had glaucoma and nonproliferative diabetic retinopathy presented with complaint of blurred vision in both eyes. Snellen visual acuity was 0.6 and vitreomacular traction (VMT) was detected on spectral domain optical coherence tomography in the right eye (A). There was highly elevated serous macular detachment and epiretinal membrane in association with VMT with a visual acuity of 0.05 in the left eye (E). Pars plana vitrectomy was performed on the left eye. The macula gradually reattached after surgery and final visual acuity was 0.2 in the left eye (F, G). During follow-up, the right eye was observed for the first 3 months and traction was seen to progress with an associated visual acuity decrease to 0.4 (B). Pneumatic vitreolysis with pure SF6 resulted in VMT release the next day (C). Visual acuity increased to 0.7 at final visit 24 months after treatment (D)

Examination of fellow eyes revealed vitreomacular interface (VMI) disorders in 8 of 12 patients (Table 1). Five patients had VMT in the fellow eye, and VMT had resolved spontaneously in 2 eyes of 2 of those patients. Both eyes of patient 6 had VMT and were included in our study for PV treatment. PPV had been performed previously for the treatment of VMT causing total macular detachment in one patient and another one is still being followed up (Figure 3). One patient previously underwent PPV surgery for FTMH in the fellow eye. One patient had epiretinal membrane in the fellow eye.

A horseshoe retinal tear was detected at the 5 o’clock position in the equatorial area 5 days after pneumatic vitreolysis and was treated with laser photocoagulation in Patient 1. Intravitreal gas (C3F8) migrated into the anterior chamber during the procedure in another patient, who was phakic. The gas could be partially removed by anterior chamber paracentesis and caused no further complications. No other complication such as endophthalmitis or cataract progression was seen. Cataract progression can be reduced by avoiding supine position in order to prevent contact between gas and lens.

## Discussion

There is a consensus about observing patients with VMT for a few months before initiating any treatment, because spontaneous VMT release is not uncommon. Nevertheless, longstanding cases may lead to the formation of ERM; therefore, the timing of treatment is still questionable.^23^

This study presents our results of PV with C3F8 and SF6 gases with “drinking bird” head movements for the treatment of VMT syndrome with 100% release rate within a mean duration of 5.2 days.

PV was first described by Chan et al.^18^ in 1995 (pre-OCT era) with complete PVD in 18 of 19 eyes (94.7%). Total PVD was achieved with 0.3-0.5 mL intravitreal C3F8 injection in 2-9 weeks (average 4 weeks) and B-scan ultrasonography was used for the PVD evaluation. Jorge et al.^19^ reported similar results of PVD induction with C3F8. Rodrigues et al.^21^ Yu et al.^24^ and Steinle et al.^22^ reported VMT release rates of 40%, 87.5% and 73% at 1 month with C3F8, respectively. Chan et al.^25^ recently reported the largest series on PV with C3F8 and achieved successful PVD in 86% of 50 eyes at a median of 3 weeks. Although numerous studies have demonstrated the efficacy of C3F8 in PV, there are fewer studies in the literature regarding SF6, which has also been used for PV, with lower and delayed release rates.^26^ Mori et al.^26^ reported that 19 of 20 patients had total PVD following PV with SF6, confirming our results. They instructed patients to keep their head in prone position during the first 3-5 days after PV and achieved PVD induction in 2 weeks. Day et al.^27^ recently reported 55.6% VMT release using PV with SF6. The procedure did not include positioning in their study, which may explain their lower release rates compared to other studies.

In our study, C3F8 was used in the 2 eyes with FTMH and 2 of the eyes with VMT, while the other 9 eyes with VMT received SF6. We used both gases to understand whether there was a difference in VMT release pattern and time. We observed 100% VMT release rate with both gases and there was no difference between them in terms of time to VMT release after the procedure. A shorter duration gas may be preferable for PV to eliminate the possible disadvantages of a longer acting gas like C3F8, such as increased rate of possible complications and restriction of patient’s daily activities, head positions, and mobility. Therefore, SF6 may be the first option for PV, as it has the same efficacy and the advantage of shorter duration. C3F8 may be chosen for patients with additional VMI disorders such as ERM or FTMH.

Most studies present their release rates at 1 month, but it may still be prolonged until 9 weeks; therefore, waiting for 2 months before switching to an alternative treatment has been suggested.^25,28,29^ Our average time of VMT release was 5.2 days. Initial release time was shorter in our study compared to the literature data. In most studies, face-down positioning or other maneuvers to facilitate the VMT release was not frequently applied after intravitreal gas injection. Only Steinle et al.^22^ reported high (84%) VMT release success rates with drinking bird head movements and stated that it might accelerate vitreous liquefaction and separation. On the other hand, Chan et al.^25^ reported the largest series to date with successful release of VMT in 43 of 50 eyes (86%). They instructed patients to avoid supine position and lie on one side or the stomach during sleeping hours and observed results similar to those achieved with the drinking bird maneuver. Other studies which had >80% VMT release rates used face-down posturing.^23,24^ All of our patients were instructed to bob their head forward and backward 10-20 times every 30 minutes until VMT release was detected. The possible mechanical separation effect provided by these movements may promote VMT release and shorten release time. We believe that the main reason for the complete and rapid success observed in our patient group was the addition of drinking bird head movements (Table 2). The increase in rates of VMT release over 80% with head positioning (face-down or drinking bird) suggests that posturing is crucial after PV. We believe face-down (or avoiding supine position) and drinking bird positioning have similar release rates, but that VMT release time may be shortened with drinking bird head movements 10-20 times every 30 minutes. The time to VMT release was 13 days in Steinle’s study^22^ and 5.2 days in our study. Mean VMT release time was longer in the other studies which did not use posturing or used only face-down positioning (Table 2).

All of our patients (13 of 13 eyes) had focal adhesion (≤1500 µm). The mean of HVMA in VMT patients was 369 µm (range: 64-630 µm). Our study results together with the current evidence in the literature suggest that having a focal VMA size close or under 500 µm seems to be essential to obtain good results in VMT syndrome.^21,22,25,27^ Rodrigues et al.^21^ previously defined three criteria that predicted treatment failure with 100% certainty: 1) HVMA ≥750 µm; 2) central foveal thickness ≥ 500 µm; and 3) moderate or high posterior hyaloid reflectivity. Foveal thickness and HVMA measurements were below these criteria in all of our VMT patients, but unfortunately we did not analyze vitreous face reflectivity.

OCT has increased our knowledge about VMA and the detection of VMI disorders.^30,31^ OCT measurements can be used as a predictor of successful treatment and possible visual acuity increase.^21,32,33^ Rodrigues et al.^21^ reported that VMT release with PV increased in patients with low posterior hyaloid reflectivity on OCT. Sun et al.^32^ determined that resolution of cone outer segment tips line and inner segment/outer segment line defects observed on SD-OCT was positively correlated with visual acuity improvement after VMT treatment with PV. SD-OCT based studies also showed that fellow eyes of patients with VMT or FTMH are at increased risk of developing VMI disorders.^34,35,36,37^ It is important to examine the fellow eye and follow up with OCT. In our study, 8 of 12 patients had VMI disorders such as VMT, FTMH, and ERM. Five patients had VMT in their fellow eye, which was also a candidate for PV. One of our patients had bilateral VMT which was treated with PV 4 days apart (Figure 1).

The PV technique is also used for the treatment of stage 2 macular holes. Chan et al.^18^ reported a 50% closure rate of FTMH with intravitreal injection of C3F8 in 1995. Jorge et al.^19^ observed successful FTMH closure in 5 of 6 eyes with 0.4 mL intravitreal C3F8. Mori et al.^26^ reported that 19 of 20 patients had total PVD and 50% of patients with FTMH had anatomical closure of the hole with SF6 injection alone. Chan et al.^25^ recently reported a 100% VMT release rate in eyes with FTMH but the hole closure rate was only 53% with one injection of C3F8. We observed rapid VMT release in 2 patients with small FTMH with VMT, but hole closure could not be achieved in either of them. Previous studies have indicated that PV may be beneficial for small FTMH with VMT.^25,26^ PPV should be the first option for the treatment of larger holes. Chan et al.^25^ suggested additional gas injections to increase the closure rate of FTMH from 53% to 67%. We did not perform any additional injection for FTMH in the present study, however. Only C3F8, which has longer duration, was used in eyes with FTMH, and patients were instructed to stay in face-down position after VMT release for a week, which was still not sufficient to close the hole in our cases (Figure 2).

Pharmacologic vitreolysis with ocriplasmin was introduced to the market with promising results compared to placebo groups.^14,15^ The MIVI-TRUST trial reported a 26.5% VMT release rate, while the OASIS study achieved 41.7% success.^14,38^ PV has higher VMT release rate (56-95%) in the literature with lower cost. Yu et al.^24^ compared PVD induction rates of ocriplasmin and PV and showed that PV had a higher VMT release rate than ocriplasmin (87.5% vs. 42.9%). Moreover, complications including transient vision loss, temporary ellipsoid zone attenuation, vitreous floaters, retinal breaks, lens subluxation, and retinal detachment have also been reported following ocriplasmin injection.^17,39,40,41^

Symptomatic VMT can be treated with PPV with a very high success rate. However, with this surgery, high cost and possible complications such as cataract, retinal tear, or endophthalmitis should always be considered.^6,12^ The PV technique has many advantages over PPV, including shorter operative time, lower cost, and eliminating the need for any kind of local or systemic anesthesia. In addition, PV complications are now well defined because of the experience with pneumatic retinopexy. Low complication rates were observed in the literature, including retinal tears, progression of VMT to FTMH, and rhegmatogenous retinal detachments.^28^ In the current study, one patient developed a peripheral retinal tear which was effectively treated with laser retinopexy. He was phakic and had high myopia. Patients that were complicated with retinal tear in the literature were also myopic and phakic patients.^28^ Attention should be paid to high myopic and phakic patients for this complication. Neither endophthalmitis nor cataract progression has been reported in the literature following PV.

## Conclusion

PV is a safe, low-cost, and relatively easier procedure than other surgical options. Consequently, for all patients with symptomatic focal VMT, in particular for older age groups with associated comorbidities, PV can be considered as first-line treatment following a certain duration of observation. Failed PV can always be followed by PPV. Limitations of this study are the limited number of patients and its retrospective nature. Further studies with more patients are needed.

## Figures and Tables

**Table 1:**
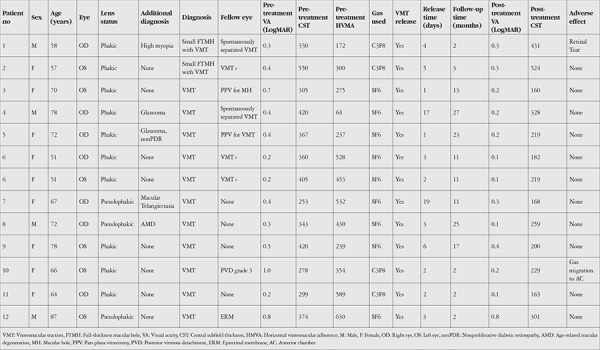
Patient demographics and characteristics before and after treatment

**Table 2:**
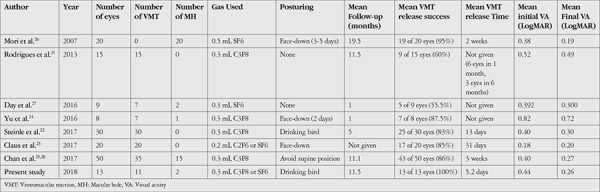
Comparison of the literature with our study
